# Retracted: Analysis of the Influence of Complexity and Entropy of Odorant on Fractal Dynamics and Entropy of EEG Signal

**DOI:** 10.1155/2018/2358107

**Published:** 2018-02-21

**Authors:** BioMed Research International

BioMed Research International has retracted the article titled “Analysis of the Influence of Complexity and Entropy of Odorant on Fractal Dynamics and Entropy of EEG Signal” [1] due to concerns about the ethical approval. An institutional investigation found that the human subjects' research was not approved by the Internal Review Board of Nanyang Technological University, where the first and last authors were affiliated.

The authors say the research was approved and conducted at Dr. Ahmadian Clinic, 3/22 St., Third Niroo Havaie Ave., Piroozi Ave., Tehran, in September 2015. This was not stated in the article and we cannot find further details of this clinic or Dr. Ahmadian. An ethical approval document for the project “Analysis of the Influence of Complexity and Entropy of Odorant on Fractal Dynamics and Entropy of EEG Signal” was provided by the first author, dated July 2, 2015, with the approval number D/A/36990 and signed by Dr. Shahaab Ahmadian. The authors also provided blank consent forms in English and Farsi. The institution asked for the article to be retracted and this is supported by the editorial board. The authors do not agree with retraction.
